# Immobilized Metal Affinity Chromatography Co-Purifies TGF-β1 with Histidine-Tagged Recombinant Extracellular Proteins

**DOI:** 10.1371/journal.pone.0048629

**Published:** 2012-10-31

**Authors:** Jasvir Kaur, Dieter P. Reinhardt

**Affiliations:** 1 Department of Anatomy and Cell Biology, Faculty of Medicine, McGill University, Montreal, Quebec, Canada; 2 Faculty of Dentistry, McGill University, Montreal, Quebec, Canada; Hospital for Sick Children, Canada

## Abstract

Extracellular recombinant proteins are commonly produced using HEK293 cells as histidine-tagged proteins facilitating purification by immobilized metal affinity chromatography (IMAC). Based on gel analyses, this one-step purification typically produces proteins of high purity. Here, we analyzed the presence of TGF-β1 in such IMAC purifications using recombinant extracellular fibrillin-1 fragments as examples. Analysis of various purified recombinant fibrillin-1 fragments by ELISA consistently revealed the presence of picomolar concentrations of active and latent TGF-β1, but not of BMP-2. These quantities of TGF-β1 were not detectable by Western blotting and mass spectrometry. However, the amounts of TGF-β1 were sufficient to consistently trigger Smad2 phosphorylation in fibroblasts. The purification mechanism was analyzed to determine whether the presence of TGF-β1 in these protein preparations represents a specific or non-specific co-purification of TGF-β1 with fibrillin-1 fragments. Control purifications using conditioned medium from non-transfected 293 cells yielded similar amounts of TGF-β1 after IMAC. IMAC of purified TGF-β1 and the latency associated peptide showed that these proteins bound to the immobilized nickel ions. These data clearly demonstrate that TGF-β1 was co-purified by specific interactions with nickel, and not by specific interactions with fibrillin-1 fragments. Among various chromatographic methods tested for their ability to eliminate TGF-β1 from fibrillin-1 preparations, gel filtration under high salt conditions was highly effective. As various recombinant extracellular proteins purified in this fashion are frequently used for experiments that can be influenced by the presence of TGF-β1, these findings have far-reaching implications for the required chromatographic schemes and quality controls.

## Introduction

Synthesis of recombinant extracellular proteins in the human 293 embryonic kidney cell (HEK293) expression system enables appropriate folding and post-translational modifications, thus generating secreted proteins that are functional and structurally similar to the native molecules [1], [2], [3], [4], [5], [6]. To purify the recombinant protein, a common technique includes the addition of a poly-histidine tag at either terminus of the protein; this small tag does typically not alter protein conformation and the imidazole functional group on histidine residues allows for coordination with divalent metal ions and thus purification by immobilized metal affinity chromatography (IMAC) [7]. The histidine-tagged protein binds to nickel and other transition metals immobilized on either an imminodiacetic acid or nitrilo-tri-acetic acid modified chromatography column with high affinity, whereas protein contaminants without the histidine-tag bind weakly or not at all [8]. Histidine-tagged proteins are eluted with imidazole in the range of 20–200 mM, which competitively displaces proteins bound to the immobilized metal ions [9]. This scheme is widely used to generate numerous secreted proteins including stroma proteins, basement membrane proteins, matricellular proteins, blood proteins and signaling molecules [10], [11], [12], [13], [14]. The purity of the recombinant protein is typically assessed by SDS-PAGE, Western blotting and mass spectrometry. However, this quality control may be insufficient when the objective of the study is to investigate cell signaling pathways regulated by the protein of interest. In this study, we tested the hypothesis that potent signaling molecules of the TGF-β superfamily are co-purified in this general purification scheme using extracellular fibrillin-1 as an example. If this is the case, it would have important consequences for the quality control of purification schemes and for the design of experiments using recombinant secreted proteins produced in this fashion.

**Figure 1:**
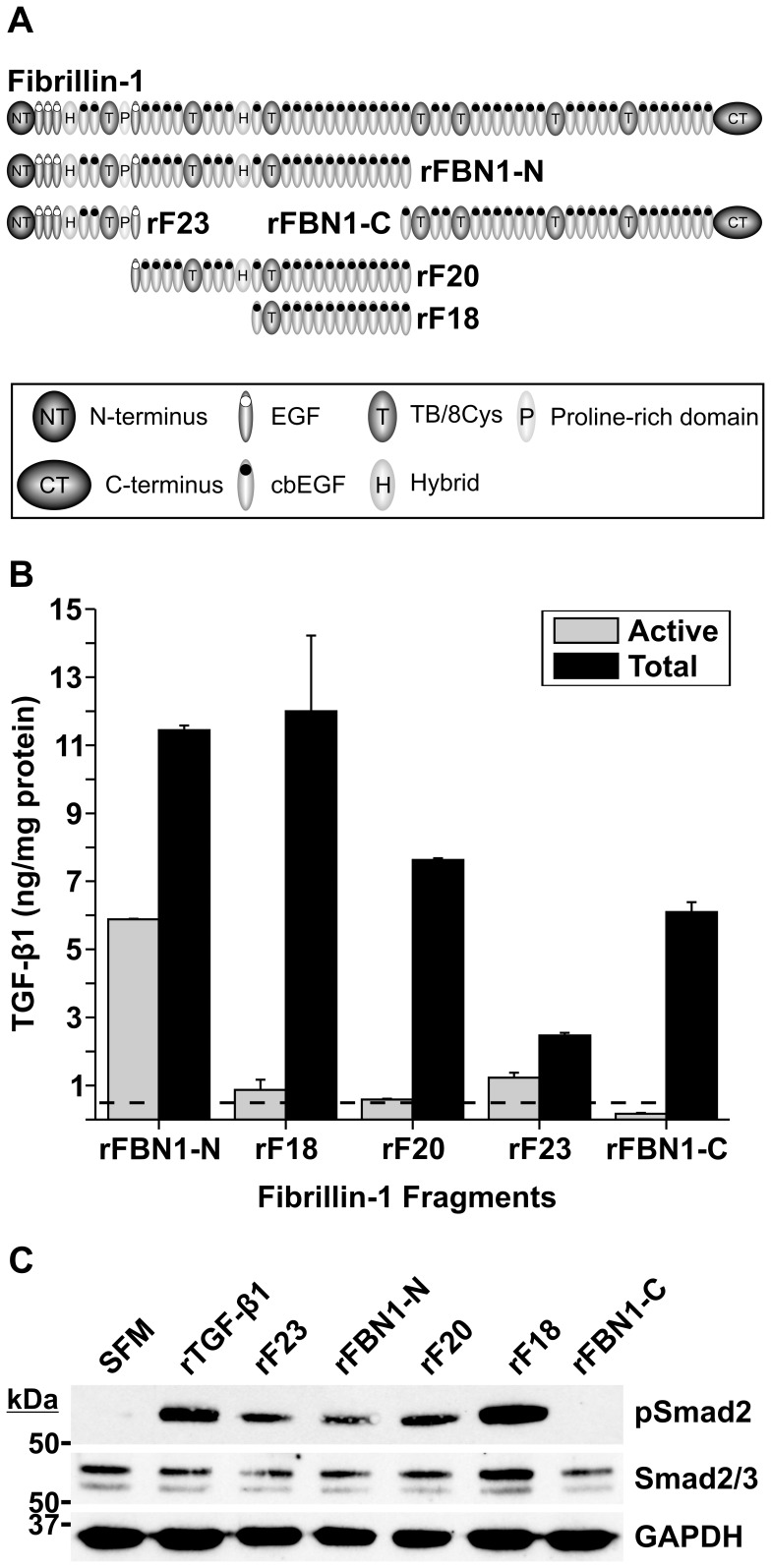
Determination of TGF-β1 in preparations of recombinant fibrillin-1 fragments. A: Schematic overview of recombinant fibrillin-1 fragments used in this study. **B:** A sandwich ELISA was used to measure levels of active (grey bars) and total (black bars) human TGF-β1 present in samples of purified recombinant fibrillin-1 fragments. In order to activate latent LAP-TGF-β1 into immunoreactive active TGF-β1, the fragments were acidified with 1 M HCl for 10 min (see Materials and Methods). This enabled the measurement of total TGF-β1 (active and latent) in purified samples of recombinant fibrillin-1 proteins. A typical result is shown. The experiment was carried out in triplicates on the same microtiter plate. Standard deviations are indicated. The coefficient of determination (r^2^) for the regression analysis of the standards used was 0.99. **C:** Cell signaling assay. The recombinant fragments were diluted to 0.4 µM in 0.2 ml of serum-free medium (SFM) and added onto MSU 1.1 cells for 1 h at 37°C. The cell lysate was analyzed by Western blotting for phospho-Smad2 (pSmad2). 4 nM of recombinant TGF-β1 (rTGF-β1) was used as a positive control, and SFM alone was included as a negative control. Total Smad2/3 and GAPDH were used as loading controls. Smad2 migrates at 60 kDa (upper band), and Smad3 migrates at 52 kDa (lower band). The positions of globular marker proteins are indicated in kDa. Note that all recombinant fragments, except rFBN1-C, trigger TGF-β1 signaling. Data in panels B and C do not correspond to the same batches of recombinant proteins. A typical result is shown.

Fibrillin-1 is a 350 kDa glycoprotein that multimerizes to form microfibril suprastructures in elastic and non-elastic tissues [15], [16], [17]. Due to its modular domain structure, fragments of fibrillins can be produced and purified conveniently as correctly folded proteins using the HEK293 expression system [18]. Direct interactions between TGF-β and fibrillin-1 have not been documented. However, other members of the TGF-β superfamily including GDF-5, BMP-2, -4, -7, and -10 can interact directly with the N-terminal region in fibrillin-1 and -2 [19], [20].

TGF-β is synthesized as a 55 kDa polypeptide that encodes the mature TGF-β at the C-terminus, and its propeptide, latency associated peptide (LAP) at the N-terminus [21]. The polypeptide homodimerizes immediately after synthesis, and undergoes proteolytic processing in the trans-Golgi network. This generates a small latency complex in which the LAP dimer is non-covalently bound to the active TGF-β dimer [22]. Association of the small latent complex with latent TGF-β binding proteins (LTBPs) promotes correct folding and secretion of TGF-β to the extracellular environment as a large latency complex [23], [24]. As LAP renders the active TGF-β dimer latent and inaccessible to the receptor, TGF-β activators such as integrins and thrombospondin-1 release TGF-β1 through conformational changes, while plasmin and matrix metalloproteinase-2, and -9 release TGF-β1 through proteolytic cleavage (for comprehensive review see Annes *et*. *al*, 2003 [25]). TGF-β signaling is transduced through type I and II transmembrane serine/threonine kinase receptors whereby ligand binding leads to phosphorylation of the type I receptor by the type II receptor [26], [27], [28]. Activation of the type I receptor activates the receptor regulated Smad proteins 2 and 3 through phosphorylation [29], [30]. This activation step triggers homo- or heterodimerization of Smad2 and 3 followed by translocation into the nucleus through association with the common Smad protein, Smad4 [29]. Smad proteins transduce TGF-β signals by either binding to specific DNA sequences or co-operating with other transcription factors [31].

In this study we show that recombinant fibrillin-1 fragments produced by HEK293 cells and purified by IMAC contain significant amounts of TGF-β1. TGF-β1 present in these protein preparations cannot be detected by Western blotting or mass spectrometry. However, TGF-β1 present in picomolar concentrations in these protein preparations has the capacity to trigger intracellular cell signaling through Smad2 and 3 proteins. We found that TGF-β secreted by HEK293 cells is not lost during ultrafiltration or IMAC, as it interacts with nickel ions immobilized to the column. A two-step purification procedure involving IMAC followed by gel filtration under high salt conditions is required to eliminate TGF-β1 from purified proteins. This finding has important implications for many studies using recombinant secreted proteins purified in this manner.

**Figure 2:**
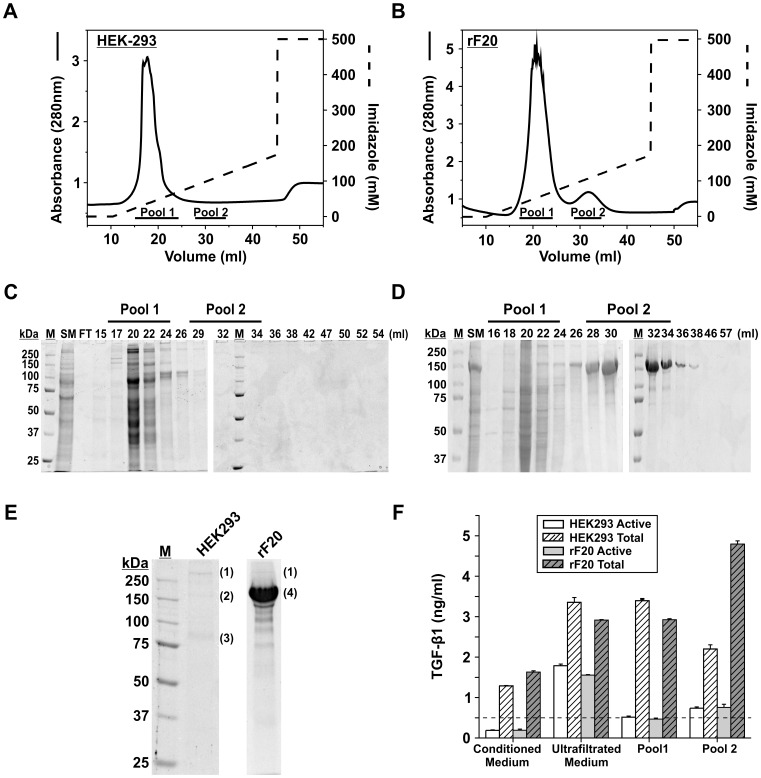
Mock and rF20 purification by IMAC. A: Chromatogram illustrating the elution profile of concentrated medium from non-transfected HEK293 cells on the IMAC column. The elution volume is indicated on the x-axis and the absorbance at 280 nm is indicated on the left-axis (black line). Protein bound to the column was displaced by an imidazole gradient as indicated (dashed line). **B:** Chromatogram illustrating the elution profile of rF20 on an IMAC column. **C:** Aliquots (20 µl) of relevant peak fractions from the mock purification were analyzed on a 7.5% SDS-PAGE and Coomassie staining (SM  =  Start Material, FT  =  flow-through). Pool 1 corresponds to weakly/non-specifically bound proteins. Pool 2 corresponds to the elution volume correlating with the elution volume for rF20 (see D, Pool 2). As the medium was from non-transfected HEK293 cells no protein peak at OD_280 nm_ occurred in the absence of a strongly expressed recombinant protein. **D:** Aliquots (20 µl) from relevant peak fractions were analyzed from the rF20 purification on a 7.5% SDS-PAGE and Coomassie staining. **E:** 20 µl of Pool 2 from the mock and the rF20 purification visualized by colloidal Coomassie staining on a 7.5% gel under reducing conditions. Pool 2 rF20 was overloaded in order to identify minor bands. Four bands labeled (1), (2), (3) and (4) were excised and analyzed by MS/MS. They are identified as tenascin C (1), keratin-1 (2) keratin-9 (3) and fibrillin-1 (4). M indicates globular marker proteins in kDa. **F:** The concentrations of TGF-β1 in ng/ml present at every step of the purification scheme was determined by an ELISA (r^2^ = 0.982). Conditioned medium refers to medium collected from confluent cell layers. Concentrated medium is the concentrate of the ultrafiltration that is loaded onto the IMAC column. Plain bars represent active TGF-β1 present in the mock (white) and rF20 (light grey) purification at different stages. The dashed bars illustrate total TGF-β1 (active and latent) present in the mock (white) and rF20 (dark grey) purifications. The threshold level for reliable readouts is indicated by a dashed line.

**Figure 3:**
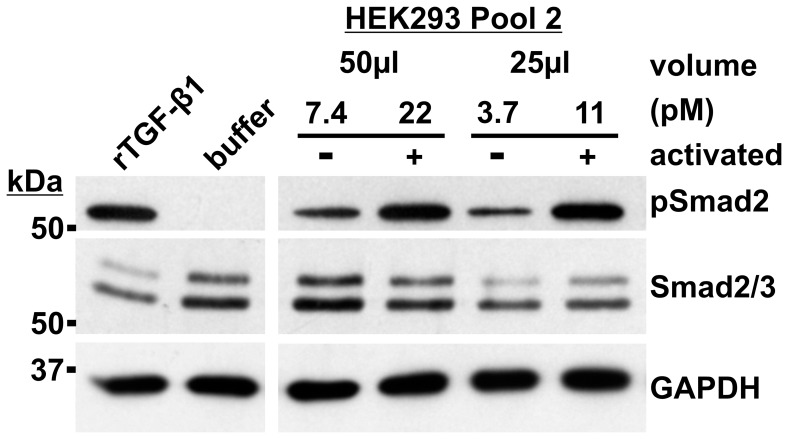
Cell signaling assay with Pool 2 of the mock purification. 50 µl of Pool 2 of the mock purification (see Fig. 2A) was titrated two-fold by serial dilutions in 0.2 ml of SFM, and added on MSU 1.1 cells for 1 h at 37°C. LAP-TGF-β1 was activated (+) by acidification (1 M HCl, 10 min). Concentrations of active and total TGF-β1 are indicated in pM. The negative control is 50 µl of TBS/Ca treated with 1 M HCl for 10 min and diluted in 0.2 ml of SFM. 4 nM rTGF-β1 diluted in SFM is included as the positive control. The cell lysate was analyzed by Western blotting for pSmad2 (12 µg of total protein), total Smad2/3 (12 µg of total protein), and GAPDH (5 µg total protein); the latter two are loading controls. The positions of the globular marker proteins are indicated in kDa.

## Materials and Methods

### Cell Culture

Human skin fibroblasts (HSFs) were isolated from the foreskin of healthy individuals (2–5 years old) after a standard circumcision procedure. The local ethics committee approved this procedure (PED-06-054), and foreskin was obtained with the consent of the donor’s parents. HSFs were used between passages 4–8, and cultured in Dulbecco’s modified Eagle’s medium (DMEM; Wisent) supplemented with 10% fetal calf serum (FCS; Wisent), 100 µg/ml penicillin, 100 µg/ml streptomycin, and 2 mM glutamine (PSG) at 37°C in a 5% CO_2_ atmosphere. The human fibroblast cell line, MSU 1.1, was cultured under identical conditions and was used in some cell signaling assays as indicated in the relevant figures [32]. This cell line has some advantages over primary HSFs including a higher proliferation rate, while not compromising matrix protein secretion and assembly [33]. In addition, pretests demonstrated that HSFs and MSU 1.1 cells trigger similar levels of Smad2 phosphorylation when stimulated with TGF-β1 in a dose-dependent manner (data not shown).

**Figure 4:**
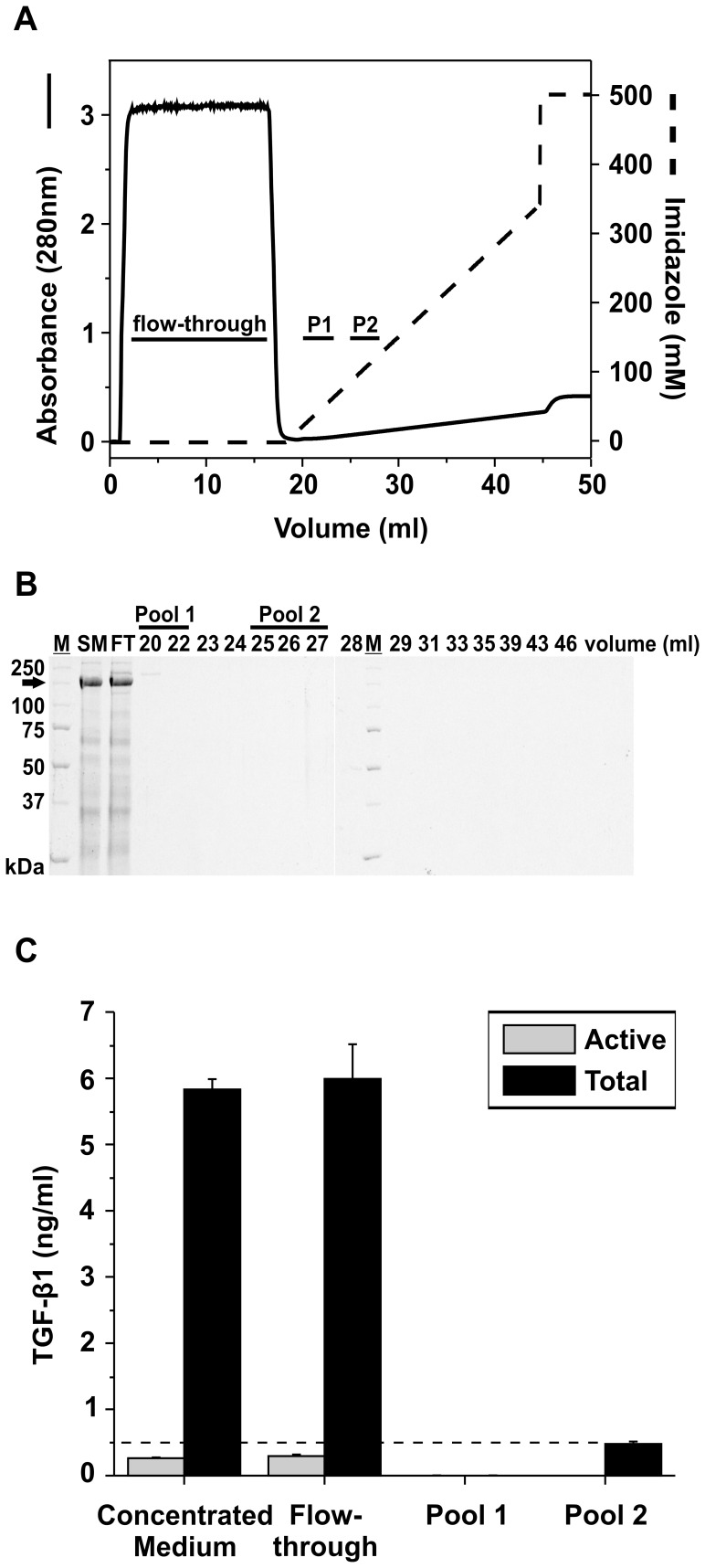
Pre-absorption chromatography. Concentrated medium containing rFBN1-N was loaded onto an IMAC column without pre-loaded nickel ions. **A:** The chromatogram displays the purification profile of rFBN1-N on the empty IMAC column. The left-axis (black line) displays absorbance at 280 nm. The x-axis denotes elution volume and right-axis (dashed black line) indicates the imidazole gradient. Pool 1 (P1, 20–22 ml) and Pool 2 (P2, 25–27 ml) indicate the volume (ml) at which protein contaminants and homogenous recombinant protein elute respectively during a standard IMAC purification of rFBN1-N. **B:** Aliquots (20 µl) from relevant peak fractions were analyzed from the rFBN1-N purification in A on a 7.5% SDS-PAGE under reducing conditions and stained with Coomassie Brilliant Blue. SM = start material, FT  =  flow-through, and M indicates globular marker proteins in kDa. **C:** TGF-β1 sandwich ELISA with fractions from the Pool 1 and Pool 2 of pre-absorption chromatography (r^2^ = 0.991). The experiment was carried out on the same microtiter plate. The threshold level for reliable readouts is indicated by a dashed line. The data represents mean of duplicates, and standard deviations are indicated.

The plasmid vectors to express the recombinant fibrillin-1 fragments (rFBN1-N, rFBN1-C, rF18, rF20, and rF23) were stably transfected into HEK293 as described previously [1], [34], [35]. Transfected HEK293 cells were cultured to confluency in 8 triple-layer flasks of 500 cm^2^ surface areas each (Nunc International) in DMEM supplemented with 10% FCS, PSG and 250 µg/ml of gentamicin sulfate (Wisent) to maintain clonal selection. Cells were washed twice with 65 ml of 20 mM HEPES, 150 mM NaCl, 2.5 mM CaCl_2_, pH7.4 to remove serum proteins, and cultured in serum-free DMEM (SFM). 500 ml of conditioned medium was collected every 48 h over a period of four weeks. The conditioned medium was centrifuged at 6,000×g for 15 min at 4°C in order to remove cellular debris, and was supplemented with 100 µM phenylmethanesulfonylfluoride (PMSF) to prevent protein degradation by serine proteases. The medium collections were stored at −20°C until used for protein purifications.

**Figure 5:**
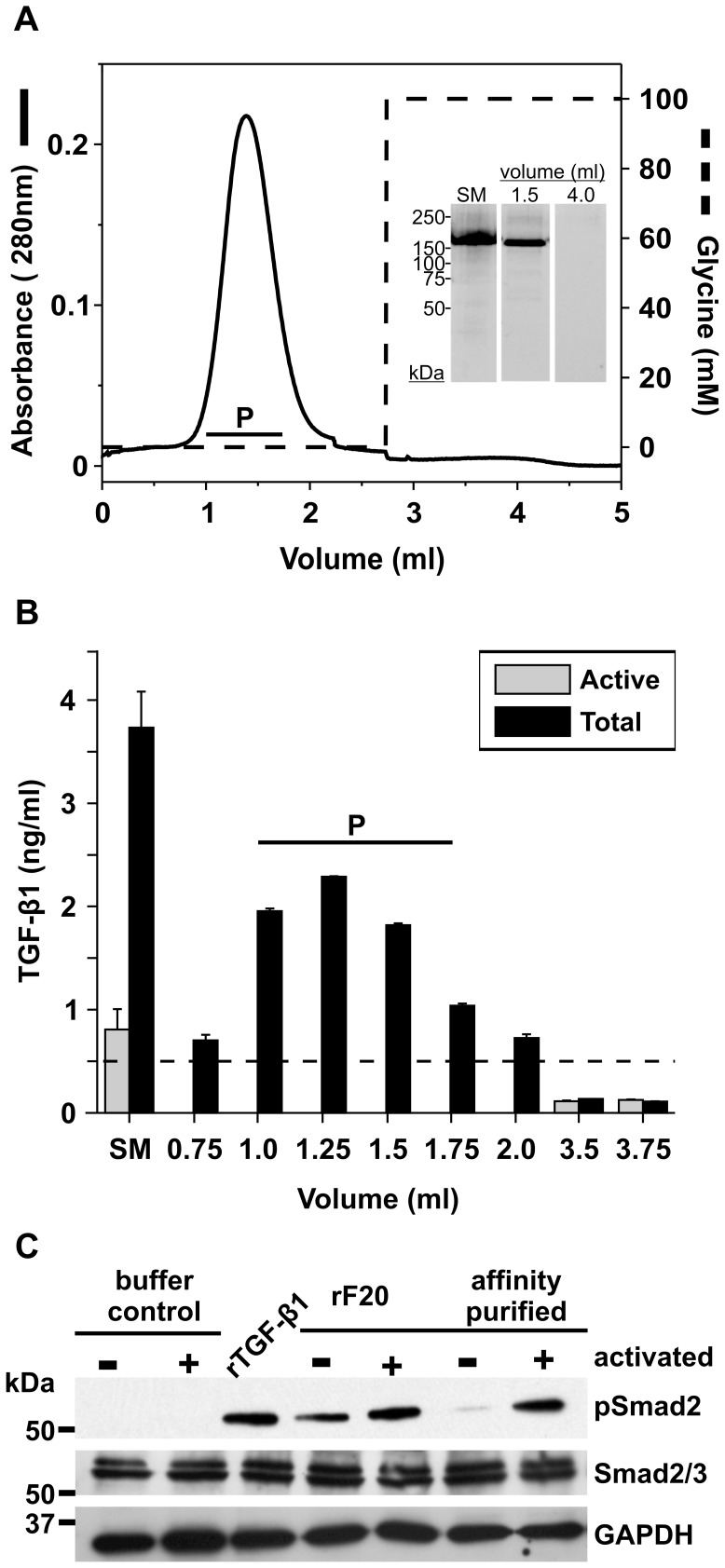
TGF-β1 antibody affinity chromatography of rF20. Recombinant rF20 was subjected to antibody affinity chromatography using an immobilized anti-TGF-β1 antibody (see Materials and Methods). **A:** The chromatogram illustrates the elution volume (x-axis, ml) versus the absorbance measured at 280 nm (left-axis, black line). TGF-β1 is eluted from the column with 0.1 M glycine-HCl, pH3 (right-axis, dashed line). The inset demonstrates aliquots (20 µl) of representative peak fractions separated on a 4–12% SDS-PAGE under reducing conditions, and analyzed by AgNO_3_ staining. Positions of globular marker proteins (M) are indicated in kDa; SM = start material. **B:** A sandwich ELISA was used to assess levels of TGF-β1 present in the rF20 fractions before and after antibody affinity chromatography. The r^2^ for the standard curve was 0.99. Latent LAP-TGF-β1 was activated by acidification in to active TGF-β1. This enabled the measurement of total TGF-β1 (active and latent) in the start material (SM) and eluted fractions (ml). The threshold level for reliable readouts is indicated by a dashed line. The experiment was carried out in duplicates on the same microtiter plate. Standard deviations are indicated. **C:** Cell signaling assay. 50 µg/ml (0.4 µM) of standard-purified (IMAC) and in addition affinity-purified rF20 was added onto HSFs for 1 h at 37°C. LAP-TGF-β1 was activated (+) into active TGF-β1 by acidification. Buffer control corresponds to TBS/Ca diluted in SFM. 4 nM of rTGF-β1 is included as the positive control. The cell lysate was analyzed by Western blotting for pSmad2 (12 µg total protein), total Smad2/3 (12 µg total protein), and GAPDH (5 µg total protein). The positions of the globular marker proteins are indicated in kDa.

#### 2.2. Chromatographic procedures

All chromatographic procedures were performed using an ÄktaPurifier 10 low pressure liquid chromatography system (GE Healthcare).

#### IMAC

Recombinant fibrillin-1 fragments were purified to homogeneity by IMAC in the following manner: 2.5 L of conditioned medium was concentrated via pressurized ultrafiltration using 30 kDa cutoff ultrafiltration membranes (Millipore) in stirred cells (Amicon) at 4°C. The concentrated conditioned medium was dialyzed twice over 24 h against 2 L of 20 mM HEPES, 500 mM NaCl, pH7.4 (running buffer) at 4°C. To eliminate any remaining debris, the concentrate was centrifuged at 10,000×g for 15 min at 4°C. The supernatant was transferred to a 50 ml superloop (GE Healthcare), and passed at 0.5 ml/min through a 5 ml Histrap HP column (GE Healthcare) preloaded with nickel ions and equilibrated in running buffer. The bound protein was eluted at 1 ml/min with a 0–200 mM linear imidazole gradient followed by 500 mM imidazole in 20 mM HEPES, 500 mM NaCl, pH7.2. Fractions containing the purified protein as assessed by Coomassie Blue staining were pooled, supplemented with 2 mM EDTA to remove traces of nickel ions and dialyzed twice against 1 L of Tris-buffered saline (TBS, 50 mM Tris-HCl, 150 mM NaCl, pH7.4) containing 2 mM CaCl_2_ (TBS/Ca) over a 24 h period at 4°C.

To test whether TGF-β1 can bind to a nickel-loaded IMAC column, 1.5 µg of active TGF-β1 (Peprotech; product #100-21) was diluted in 0.12 ml of running buffer and loaded at 0.01 ml/min onto a 1 ml nickel-loaded Histrap HP column equilibrated in running buffer. Bound protein was eluted at 1 ml/min with a 0–300 mM linear imidazole gradient followed by 500 mM imidazole in 20 mM HEPES, 500 mM NaCl, pH7.2. Aliquots (0.1 ml) of eluted fractions (1 ml) were analyzed for TGF-β1 as described below. Similarly, to analyze the ability of LAP to interact with the column material, 5 µg of LAP (R&D Systems; product #246-LP/CF) was diluted in 0.25 ml of running buffer and loaded onto the column under identical conditions. Aliquots (0.1 ml) of eluted fractions (1 ml) were analyzed by a direct ELISA for LAP (see below). Neither recombinant LAP nor recombinant TGF-β1 contain histidine-tags.

**Figure 6:**
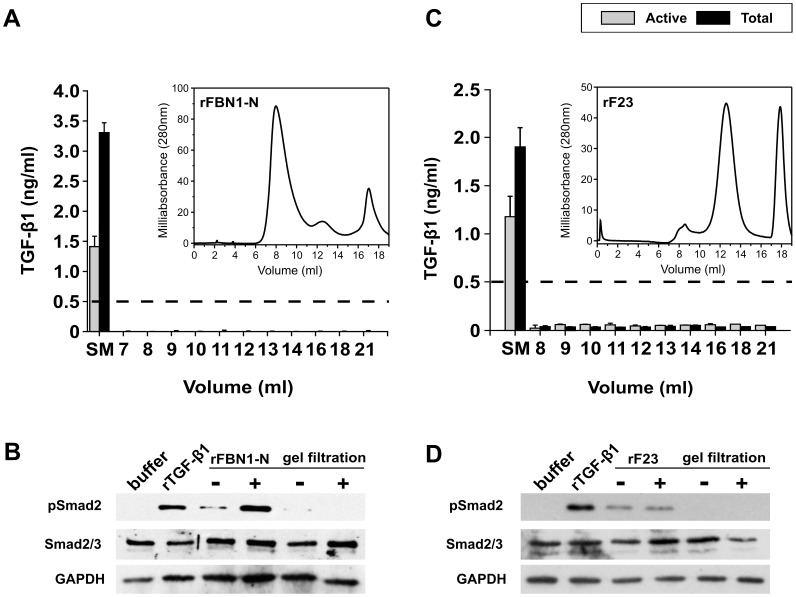
Gel filtration chromatography of rFBN1-N and rF23. Fibrillin-1 fragments rFBN1-N and rF23 were subjected to gel filtration chromatography on an analytical Superose 12 column equilibrated with 50 mM Tris-HCl, 800 mM NaCl, pH7.4. **A:** TGF-β1 present in the rFBN1-N preparation before and after gel filtration was quantified by a sandwich ELISA. The r^2^ for the standard curve was 0.981. To measure total TGF-β1 in the start material (SM) and eluted fractions (ml), latent LAP-TGF-β1 was activated by acidification into immunoreactive active TGF-β1. The experiment was carried out in triplicates on the same microtiter plate. Standard deviations are indicated. The threshold level for reliable readouts is indicated by a dashed line. The inset illustrates the elution profile of rFBN1-N at OD**_280_**_**nm**_. The major protein peak is at 8 ml while the peak at 17 ml reflects protein trailing, which is typical for recombinant fragments encompassing the N-terminal half of fibrillin-1. **B:** Cell signaling assay. HSFs were incubated with 0.4 µM (56.4 µg/ml) of rFBN1-N prior to and after gel filtration chromatography for 1 h at 37°C. LAP-TGF-β1 was activated (+) into active TGF-β1 by acidification. The buffer control refers to TBS/Ca treated with 1 M HCl for 10 min, and diluted in 0.2 ml of SFM. 4 nM of rTGF-β1 is included as the positive control. The cell lysate was analyzed by Western blotting for pSmad2 (12 µg of total protein), total Smad2/3 (12 µg of total protein), and GAPDH (5 µg of total protein). **C:** The ELISA results quantify TGF-β1 amounts present in the rF23 preparation before (start material, SM) and after gel filtration (represented by elution volume, ml). Experiment carried out in triplicates, and standard deviations are indicated (r^2^ = 0.984). The inset exhibits the elution profile of rF23. The major protein peak is at 12.5 ml while the peak at 17.8 ml reflects protein trailing. **D:** Cell signaling assay. HSFs were incubated with 0.4 µM (20 µg/ml) of the start material and the gel filtration purified rF23 for 1 h at 37°C.

### Antibody Affinity Chromatography

For TGF-β1 antibody affinity chromatography, the column material was generated by coupling 0.5 mg of TGF-β1 antibody (R&D Systems; product #MAB240) to cyanogen bromide (CNBr) activated sepharose 4B (GE Healthcare; product #17-0430-01) according to the manufacturer’s instructions. The TGF-β1 antibody column was equilibrated in TBS/Ca, pH7.4. 0.3 mg of rF20 containing TGF-β1 was loaded onto the column at a flow rate of 0.01 ml/min, and 0.25 ml fractions were collected. Antibody-bound TGF-β1 was eluted with 0.1 M glycine-HCl, pH3.0 at 0.25 ml/min and immediately neutralized with 1 M Tris. Aliquots (20 µl) of selected peak fractions were heat-denatured (95°C for 3.5 min), and separated by SDS-PAGE under reducing conditions (20 mM dithiothreitol, DTT). The protein bands were visualized by standard AgNO_3_ staining [36].

**Figure 7:**
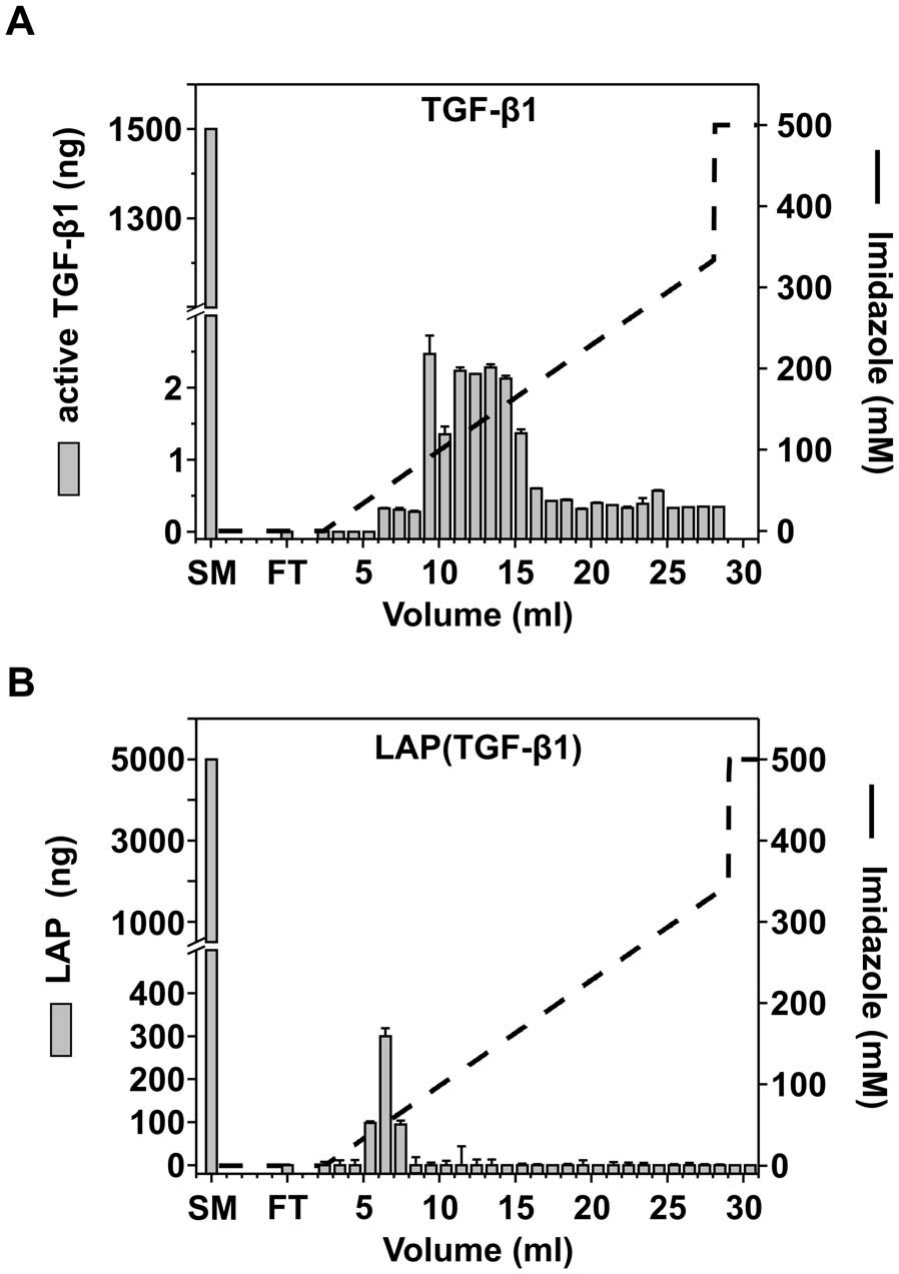
IMAC of recombinant TGF-β1 and LAP. **A:** 1.5 µg of active TGF-β1 was loaded onto a 1 ml nickel-charged IMAC column. The x-axis denotes the elution volume (ml), and the left-axis denotes the amount of active TGF-β1. The right-axis (dashed line) illustrates the imidazole gradient. No protein peak was detected at OD**_280_**_**nm**_ (data not shown). The grey bars indicates active TGF-β1 (ng) present in the start material (SM), flow-through (FT) and eluted fractions (volume, ml), determined by a sandwich ELISA for TGF-β1. The experiment was carried out in triplicates, and standard deviations are indicated (r^2^ = 0.99). **B:** 5 µg of LAP (TGF-β1) was loaded onto a 1 ml nickel-charged IMAC column. The grey bars indicate the amount of LAP (ng) detected in the SM, FT and eluted fractions by a direct ELISA. The data represents mean of duplicates and standard deviations are indicated (r^2^ = 0.99).

### Gel Filtration Chromatography

For gel filtration chromatography, a Superose 12 column (GE Healthcare; 23 ml total volume) was equilibrated with 50 mM Tris-HCl, 800 mM NaCl, pH7.4. 0.5 mg of protein was loaded onto the column at a flow rate of 1 ml/min, and 0.5 ml fractions were collected. Aliquots (20 µl) of the fractions were separated under reducing conditions by 7.5% SDS-PAGE, followed by AgNO_3_ staining. Fractions containing recombinant protein were pooled and dialyzed overnight against 1 L of TBS/Ca at 4°C. The protein was stored at -20°C, and further used in cell signaling assays.

### Sandwich ELISA (TGF-β1)

The amount of TGF-β1 present in various purified fibrillin-1 fragment preparations was determined by ELISA (R&D systems; product #DY240), and performed on 96-well microtiter plates (Maxisorp; Nalge Nunc International) at 22°C. The capture antibody (mouse anti-human TGF-β1) was coated overnight at 2 µg/ml in phosphate buffered saline (PBS, 137 mM NaCl, 2.7 mM KCl, 10 mM Na_2_HPO_4_, 2 mM KH_2_PO_4_, pH7.4). Unbound antibody was washed off with an excess of PBST (0.05% Tween-20 in PBS). Non-specific binding sites on the surface were blocked for 2 h with 5% Tween-20 in PBS with 0.05% NaN_3_. The wells were washed with PBST and 0.1 ml of the standards and samples were incubated for 2 h. Recombinant fibrillin-1 fragments were diluted 4-fold to approximately 0.1 mg/ml in TBS/Ca and then added onto a microtiter plate. To determine total TGF-β1 (active + latent) present in fibrillin-1 protein preparations, TGF-β1 was dissociated from LAP by adding 0.02 ml of 1 M HCl (∼pH1, not adjusted) to 0.1 ml volume of protein for 10 min at 22°C. The acidification was neutralized with 0.02 ml of 0.5 M HEPES, 1 M NaOH (∼pH14, not adjusted). TGF-β1 bound to the capture antibody was detected with a biotinylated chicken anti-human TGF-β1 antibody. The detection antibody was diluted to a working concentration of 0.3 µg/ml in PBST containing 14 mg/ml delipidized bovine serum albumin (BSA, R&D systems; product #DY997). After 2 h of incubation with the detection antibody, the wells were washed and incubated with streptavidin-horse radish peroxidase (HRP) for 20 min. After washing unbound streptavidin-HRP, the color development reaction was carried out with 0.1 ml of ready-to-use peroxidase substrate containing 3,3,5,5′-tetramethylbenzidine (TMB) for 20 min (Sigma Aldrich; product #T0440), and stopped with 0.05 ml of 2N H_2_SO_4_. The absorbance was measured at 450 nm using a Beckman Coulter microplate reader (model number AD 340). The standard, recombinant human TGF-β1 was serially diluted two-fold from 2000 pg/ml to 125 pg/ml in TBS/Ca. The standard curve was generated by plotting the absorbance at 450 nm of each standard on the y-axis against the concentration (pg/ml) on the x-axis. Linear regression analysis was performed from these standard concentrations and used to determine unknown TGF-β1 concentrations present in the samples. The detection limit for the ELISA was determined at 500 pg/ml because the absorbance values obtained from TGF-β1 standards below this concentration are close to background values generated by buffer alone. This 500 pg/ml threshold value is indicated in all measurements presented.

### Direct ELISA (LAP)

Recombinant LAP and eluted fractions (0.1 ml) from IMAC of LAP were coated overnight at 4°C on 96-well microtiter plates (Maxisorp; Nalge Nunc International). Immobilized LAP was blocked for 1.5 h at 22°C with 0.3 ml of 30 mg/ml delipidized BSA, 0.1% NaN_3_ in 0.05% TBST (0.05% Tween-20 in TBS). Wells were washed four times with an excess of 0.05% TBST, and then incubated for 2 h with 0.5 µg/ml of LAP antibody (R&D Systems; product #AF-246-NA) in 0.05% TBST containing 1 mg/ml delipidized BSA and 0.1% NaN_3_. Unbound antibody was removed by four washes with 0.05% TBST and then incubated for 1 h with donkey anti-goat-HRP antibody (Invitrogen) 1∶3,000 diluted in 0.05% TBST containing 1 mg/ml delipidized BSA and 0.1% NaN_3_. The reaction was developed with the addition of 0.1 ml TMB for 30 min, and stopped with 0.05 ml of 2N H_2_SO_4_. The standard, LAP, was serially diluted two-fold from 2000 pg/ml to 31.25 pg/ml in 0.1 ml of 20 mM HEPES, 500 mM NaCl, 500 mM imidazole, pH7.2. After linear regression analysis of these standard concentrations unknown LAP concentrations were determined.

### Tandem Mass Spectrometry Analysis

10 µg of protein was separated on a 7.5% SDS-PAGE under reducing conditions, and stained with Coomassie Brilliant Blue G-250 colloidal protein stain (Sigma Aldrich; product #BP100-25). The bands were excised and subjected to a standard trypsin digestion (12 ng trypsin/µl gel volume). The peptide digest was analyzed on a Brucher HCT Ultra ion trap mass spectrometer using an electron spray ionization source. The data files generated were compared with the mammalian NCBI database (version NCBInr 20110411) using Mascot V.2.2 (Matrix Sciences).

### Cell Signaling Assay

The cell signaling assay was used to test the signaling capability of TGF-β1 present in various fibrillin-1 fragment preparations in either MSU 1.1 cells or HSFs. 3×10^5^ cells were seeded in 24-well culture plates (Corning Costar) and cultured for 3d. The cells were washed twice with 0.5 ml of SFM and incubated with 0.5 ml of SFM for 16 h to remove any growth factors remaining from the cultivation in serum-containing medium. The recombinant fibrillin-1 fragment preparations were diluted to 0.4 µM in 0.2 ml of SFM. In absolute concentrations this corresponds to 50 µg/ml of rF20, 27 µg/ml of rF18, 54.5 µg/ml of rFBN1-C, 65.6 µg/ml of rFBN1-N, and 20 µg/ml of rF23. The fragments were added onto MSU 1.1 cells or HSFs for 1 h at 37°C. This incubation period was determined by pretests in which fragments were incubated for 15 min, 60 min and 90 min (data not shown). The strongest Smad2 phosphorylation was observed at 60 min, and this incubation period was used for all subsequent cell signaling assays. As a positive control, 4 nM of recombinant TGF-β1 (R&D Systems, product #240-B) was diluted in 0.2 ml of SFM. In order to obtain a maximum signal, TGF-β1 was added in amounts above physiological levels. After 1 h incubation, the cells were washed twice with 0.5 ml of SFM. Cell lysis was carried out on ice with 0.1 ml radioimmunoprecipitation assay buffer (RIPA buffer; 50 mM Tris-HCl, 1%NP-40, 150 mM NaCl, 1 mM EDTA, pH7.4) containing freshly added protease inhibitors (20 µg/ml aprotinin, 2 µg/ml leupeptin, and 1 µM PMSF) and phosphatase inhibitors (2 µM NaF, 0.5 µM sodium orthovanadate). The lysate was centrifuged at 10,000×g for 10 min at 4°C, and the supernatant was transferred into ice-cooled microcentrifuge tubes. The protein concentrations of the lysates were determined by a bicinchoninic acid assay (Thermo Scientific; product #23225) in which the BSA standards were diluted in RIPA buffer.

To analyze the lysate by Western blotting, the samples were heat-denatured (3.5 min, 95°C), and separated under reducing conditions by SDS-PAGE. To detect phospho-Smad2 (pSmad2) and Smad2/3, lysate corresponding to 12 µg of total protein were separated by 7.5% SDS-PAGE. To detect glyceraldehyde 3-phosphate dehydrogenase (GAPDH), lysates corresponding to 5 µg of total protein were separated on a 10% SDS-PAGE. The cell lysates were electrophoresed and transferred onto a 0.45 µm nitrocellulose membrane (Bio-Rad). The transfer was carried out at 0.4A in ice-cold 10 mM sodium tetraborate for 1 h. Free binding sites on the membranes were blocked with 50 mg/ml of non-fat dry milk in 0.1% TBST for 1 h at 22°C. Following 3×10 min washes with 0.2% TBST, the blots were probed with primary antibodies. Antibodies against pSmad2 (product #3101), Smad2/3 (product #3102) and GAPDH (product #2118) were purchased from Cell Signaling. The pSmad2 antibody was diluted 1∶1,000 in 50 mg/ml non-fat dry milk, 0.1% TBST. The Smad2/3 (dilution 1∶1,000 v/v) and GAPDH (dilution 1∶2,000 v/v) antibodies were diluted in 50 mg/ml BSA in 0.1% TBST. The blots were incubated overnight at 4°C. After 3×10 min washes with 0.2% TBST, membranes were incubated with secondary antibody for 1 h in the dark. The secondary antibody, goat anti-rabbit antibody conjugated with HRP (Jackson Immunoresearch Laboratories Inc.) was used at a dilution of 1∶3,500 in the same buffer as the primary antibody. The membranes were thoroughly washed for at least 3 h with 0.2% TBST to eliminate non-specific interactions, and developed by enhanced chemiluminescence (Thermo Scientific; product #E3018) on Hyblot CL autoradiography film.

## Results and Discussion

### Determination of TGF-β1 in Preparations of Recombinant Fibrillin-1 Fragments

Full length fibrillins are difficult to produce and purify due to their large size and propensity to aggregate [37]. Therefore, recombinant histidine-tagged fibrillin-1 fragments are routinely purified by IMAC [1], [38], [39]. Fibrillin-1 fragments spanning the entire molecule were analyzed for the presence of TGF-β1 by sandwich ELISA. Figure 1A schematically shows the regions encompassed by the various recombinant fibrillin-1 fragments. All recombinant proteins, except the C-terminal half fragment rFBN1-C, contained detectable levels of active TGF-β1 (Fig. 1B, grey bars). To detect additionally latent TGF-β that is normally present as LAP associated TGF-β1 (LAP-TGF-β1), the samples were acidified prior to the ELISA, resulting in readout of total TGF-β1 (Fig. 1B, active and latent, black bars). Figure 1B illustrates typical amounts of total TGF-β1 found in preparations of the central fragment rF18 (12±2.2 ng TGF-β1/mg rF18), the entire N-terminal half fragment rFBN1-N (11.4±0.14 ng TGF-β1/mg rFBN1-N), and in preparations of rF20 (7.6±0.05 ng TGF-β1/mg rF20). The C-terminal half fragment rFBN1-C contained 6±0.3 ng total TGF-β1/mg, while the N-terminal fragment rF23 contained 2.4±0.1 ng TGF-β1/mg rF23. These results were validated at least three times using different batches of each protein. While TGF-β1 was detected in every batch, we noticed batch-to-batch variations in the amount of TGF-β1. Similar TGF-β1 mRNA expression levels were observed for all HEK293 clones except those secreting rFBN1-C (Supplemental Figure S1). The presence of TGF-β1 is not restricted to preparations of fibrillin-1 fragments, as we detected significant amounts of TGF-β1 in preparations of human histidine-tagged full length fibulin-3, -4 and -5 purified by IMAC in an identical manner (data not shown). To determine if other growth factors from the TGF-β/BMP superfamily were also present, recombinant fibrillin-1 fragments were analyzed by an ELISA specific for BMP-2. Although BMP-2 is expressed by HEK293 cells and despite the fact that BMP-2 can interact with the N-terminus of fibrillin-1 [19], it was not detected in preparations of the various fibrillin-1 fragments (data not shown).

In order to test if active TGF-β1 detected in preparations of the recombinant fibrillin-1 fragments has the potential to induce Smad2 phosphorylation, equimolar amounts of the recombinant fragments rF23, rFBN1-N, rF20, rF18, and rFBN1-C were added onto fibroblasts for 1 h at 37°C. This incubation period was determined as optimal by pretests (see Materials and Methods). A typical Western blot result is demonstrated in Figure 1C. All fragments encompassing the N-terminal half of fibrillin-1 (rF23, rFBN1-N, rF18, and rF20) triggered Smad2 mediated cell signaling. The recombinant protein, rFBN1-C, encompassing the C-terminal half, was the only fragment that did not induce Smad2 phosphorylation (Fig. 1C). This correlated with and ELISA readout in Fig. 1B, which demonstrated that 97% of TGF-β1 associated with rFBN1-C is in the inactive LAP-TGF-β1 form and the remaining amount of active TGF-β1 is insufficient for stimulating intracellular signaling. This data suggests that active TGF-β1 present in rF23, rFBN1-N, rF20 and rF18 is responsible for Smad2 phosphorylation observed in the cell signaling assays. We hypothesized that the presence of TGF-β1 in the purified samples of various fibrillin-1 fragments could be due to several reasons: 1) TGF-β1 could be co-purified by the chromatographic procedures employed; 2) TGF-β1 could be bound to another protein that is in turn co-purified together with the fibrillin-1 fragments; 3) There might be a direct and specific association between TGF-β1 or LAP-TGF-β1 with the fibrillin-1 fragments. To further clarify these possibilities, the purification procedure was analyzed in detail in the following paragraphs.

### IMAC of rF20 Versus Control

The recombinant fibrillin-1 fragments were expressed and secreted by HEK293 cells, and conditioned medium harvested was routinely purified by IMAC using nickel ions. To determine if TGF-β1 in the purified protein samples is a by-product of the expression system and purification procedure, the concentration of TGF-β1 was determined by ELISA at every step of the purification scheme of fragment rF20. The TGF-β1 levels were then compared with a control, in which non-transfected HEK293 were cultured and the conditioned medium was mock-purified under identical conditions, as detailed in Materials and Methods (Fig. 2). The chromatograms illustrate the elution profile of mock HEK293 (Fig. 2A) and rF20 (Fig. 2B) purification on the IMAC column, respectively. The elution profile of the mock purification on the IMAC column shows the presence of only one peak between 15–24 ml. SDS-PAGE analysis followed by Coomassie staining detected protein contaminants primarily in fractions eluting between 15–24 ml, and negligible amounts in subsequent fractions (25–54 ml) (Fig. 2C). The elution profile of rF20 contained two peaks, which corresponded to elution volumes between 15–24 ml and 28–34 ml, respectively (Fig. 2B). Analysis of eluted fractions by SDS-PAGE showed that the first peak (15–24 ml) contained numerous protein contaminants that bound weakly to the nickel-loaded chelating column (Fig. 2D), whereas the only visible Coomassie-stained band in fractions from the second peak (28–34 ml) represented the recombinant protein rF20. For further analysis by SDS-PAGE and to compare TGF-β1 levels in the mock HEK293 and rF20 purification, fractions eluting between 15–24 ml and 28–34 ml were pooled and designated Pool 1 and Pool 2, respectively.

### Protein and TGF-β1 Analysis in Mock and rF20 Purifications

Analysis of Pool 2 of the mock and rF20 purifications by SDS-PAGE demonstrated that rF20 was purified to >90% homogeneity, whereas only three faint high molecular mass proteins (labeled 1, 2 and 3) were detected in Pool 2 of the mock purification (Fig. 2E). The protein band labeled (1) is common to both purifications. For further analysis the bands labeled (1)–(4) were excised, trypsinized and analyzed by tandem mass spectrometry (MS/MS). Band 1 was identified as the extracellular glycoprotein tenascin C, while band 2 and 3 were identified as keratin type 1 and 9, respectively. MS/MS identified band 4 unambiguously as fibrillin-1, but peptides corresponding to tenascin C were also detected. While tenascin C appears to be present in minute amounts in the rF20 preparations, it is not currently known if it is a TGF-β carrier. Analysis of the total Pool 2 of the rF20 and mock preparations by MS/MS did not detect TGF-β1, despite the ability of the rF20 preparation to trigger Smad2 phosphorylation in fibroblasts (see Fig. 1C), and despite its detectability in picomolar concentrations by ELISA (see below). Similarly, MS/MS analyses of the fragments rF23, rFBN1-N, rF18, and rFBN1-C did not identify TGF-β1. We speculated that LTBP-1, -3 and -4 and microfibril associated glycoprotein 1 (MAGP-1) are candidate proteins that could potentially co-purify with fibrillin-1 fragments. LTBPs sequester LAP-TGF-β, and can interact via their C-terminal domain with fibrillin-1 [40]. MAGP-1 binds to active TGF-β1, and can interact with fibrillin-1 at the N-terminal and central region [34], [41]. However, LTBPs and MAGP were not detected in the fibrillin-1 fragment preparations, suggesting that TGF-β1 is not present in these preparations due to co-purification of another protein that in turn binds to TGF-β1.

Figure 2F compares TGF-β1 concentrations at various stages of the mock and rF20 purification analyzed by ELISA. TGF-β1 content was measured in the following samples: conditioned medium; concentrated medium after ultrafiltration; Pool 1 and Pool 2. Conditioned medium harvested from non-transfected and rF20 expressing HEK293 cells contained 1.28±0.02 ng/ml and 1.63±0.03 ng/ml of total TGF-β1, respectively. Ultrafiltrated conditioned medium contained 1.78±0.04 ng/ml of active TGF-β1 and 3.35±0.12 ng/ml of total TGF-β1 for the mock purification. Similarly, ultrafiltrated medium containing rF20 contained 2.92±0.04 ng/ml of total TGF-β of which 1.56±0.01 ng/ml was in the active form. The ELISA did not detect active or latent TGF-β1 in the flow-through of the ultrafiltration of either purification (data not shown). Despite an ultrafiltration concentration factor of ∼100, the total TGF-β1 only concentrated by a factor of approximately 2, indicating significant losses during ultrafiltration. After IMAC, TGF-β1 eluted in picomolar concentrations in fractions containing protein contaminants (Pool 1) and the recombinant protein (Pool 2), but was not detected in the flow-through (not shown). 3.39±0.04 ng/ml of total TGF-β1 was detected in Pool 1 of the mock purification, and 2.93±0.03 ng/ml in Pool 1 of the rF20 purification. A comparable amount of active TGF-β1 was present in Pool 2 of both, the mock and rF20 purification, which contained 0.74±0.03 ng/ml and 0.75±0.08 ng/ml of active TGF-β1, respectively. Despite similar levels of active TGF-β1, there was about two-fold more total TGF-β1 associated with rF20 (4.79±0.08 ng/ml) than was present in the mock purification (2.20±0.11 ng/ml). These findings illustrate that ultrafiltration does not eliminate TGF-β1, as TGF-β1 is retained by the IMAC column. Replacing ultrafiltration with anion-exchange for concentration of the recombinant protein, resulted in similar data (data not shown). Quantification of TGF-β1 by ELISA showed that TGF-β1 and/or LAP-TGF-β1 elute together with proteins that bind to the IMAC column. These results demonstrate that TGF-β1 secreted by HEK293 cells concentrated and co-purified during IMAC.

### Cell Signaling Analysis of Pool 2 from Mock Purification

TGF-β1 detected in Pool 2 of the mock purification was tested for its cell signaling activity in fibroblasts for 1 h in a concentration dependent manner (3.7–22 pM) (Fig. 3). To activate LAP-TGF-β1, Pool 2 was acidified with HCl prior to incubation with cells. The Western blot demonstrates that TGF-β1 present in Pool 2 of the mock purification triggered Smad2 phosphorylation in a concentration dependent manner, and activation of LAP-TGF-β1 generally increased Smad2 phosphorylation. These results also illustrate that the cell signaling assay is more sensitive than the ELISA in detecting TGF-β1 levels.

### Elimination of Non-specific TGF-β1 from Purified Proteins

In order to remove co-purified TGF-β1 from fibrillin-1 preparations we used three chromatographic approaches including pre-absorption on the IMAC resin, antibody affinity chromatography and gel filtration chromatography.

#### Pre-absorption chromatography

First, we rationalized that TGF-β1 might non-specifically interact with the column resin. We pre-absorbed concentrated medium employing the IMAC resin (sepharose) in the absence of nickel ions. We tested this scheme with concentrated medium harvested from HEK293 cells expressing rFBN1-N. This recombinant protein is robustly expressed by HEK293 cells, and typically contained high amounts of total TGF-β1 (see Fig. 1B). The chromatogram in Figure 4A demonstrates the elution profile of rFBN1-N on a column without nickel ions. Proteins that did not bind to the column eluted between 1–17 ml (flow-through), and potentially bound proteins were eluted with a linear imidazole gradient. SDS-PAGE analysis of fractions of this pre-absorption chromatography illustrated as expected that rFBN1-N was present in the start material (SM) and the flow-through (FT) along with other protein contaminants but no proteins eluted in fractions between 20–46 ml (Fig. 4B). The position of Pool 1 and Pool 2 was determined by a standard purification of rFBN1-N (data not shown). Thus, fractions eluting between 20–22 ml and 25–27 ml were pooled and labeled Pool 1 and 2, respectively (Fig. 4A, P1 and P2). The presence of TGF-β1 in the various fractions of the pre-absorption step was analyzed by ELISA (Fig. 4C). Concentrated medium loaded onto the column contained 5.82±0.16 ng/ml of total TGF-β1. The ELISA did not detect TGF-β1 in Pool 1 and detected negligible amounts in Pool 2. The flow-through contained 5.98±0.52 ng/ml of total TGF-β1, which is equivalent to TGF-β1 present in the start material, demonstrating that the column resin did not non-specifically sequester TGF-β1. We conclude that the two-step pre-absorption purification technique cannot prevent co-purification of TGF-β1, and that non-specific interaction of TGF-β1 with the column resin does not represent the mechanism by which it co-purifies with recombinant proteins.

#### Antibody affinity chromatography

The second approach used to remove TGF-β1 from fibrillin-1 fragment preparations was antibody affinity chromatography. A chromatography column was generated in which a monoclonal TGF-β1 antibody was covalently coupled to CNBr-activated sepharose 4B. The elution profile of rF20 from the TGF-β1 antibody column is illustrated in Figure 5A. The peak between 1–2 ml corresponded to the flow-through, which contained rF20, as shown by analysis of the fractions by SDS-PAGE and AgNO_3_ staining (Fig. 5A, inset). At 2.75 ml, the elution of TGF-β1 bound to the antibody column was started with 0.1 M glycine, pH3.0 (Fig. 5A, dashed line). No major protein peak at OD_280 nm_ was observed in elution volumes >2.75 ml (Fig. 5A, inset). TGF-β1 quantification by ELISA demonstrated that initially rF20 contained 0.84±0.19 ng/ml of active and 3.7±0.35 ng/ml of total TGF-β1 (start material, SM) (Fig. 5B). Antibody affinity chromatography completely removed active TGF-β1 from eluted fractions containing rF20, but up to 2.33±0.01 ng/ml of LAP-bound TGF-β1 was still present at 1.25 ml elution volume. To test if affinity-purified rF20 can still induce Smad2 phosphorylation, fractions collected between 1.0–1.75 ml were pooled and exposed to fibroblasts (Fig. 5B, indicated with P). The cell signaling assay demonstrated that active TGF-β1 present in rF20 prior to antibody affinity chromatography triggered Smad2 phosphorylation, and acid activation of the LAP-TGF-β1 triggered stronger Smad2 phosphorylation (Fig. 5C). Affinity purified rF20 no longer triggered TGF-β1 signaling, unless LAP-TGF-β1 was activated. Quantification of TGF-β1 by ELISA demonstrated that rF20 contained 5±1 ng LAP-TGF-β1/mg rF20 prior to antibody affinity chromatography. Similarly, affinity-purified rF20 contained 7.5±2.5 ng LAP-TGF-β1/mg rF20. In summary, these results illustrate that the TGF-β1 antibody column completely removed active TGF-β1 that co-purified with rF20 but was not able to remove the latent fraction of TGF-β1 associated with the rF20 preparation. This suggests that the immobilized antibody is unable to access the epitope on active TGF-β1 when it is complexed with LAP.

#### Gel filtration chromatography

The third approach used to eliminate TGF-β1 from protein preparations was gel filtration chromatography under high salt concentration (0.8 M NaCl) to disrupt ionic interactions between proteins. The N-terminal half fragment rFBN1-N was subjected to gel filtration chromatography using an analytical Superose 12 column. Analysis of eluted fractions by SDS-PAGE and AgNO_3_ staining indicated that rFBN1-N eluted between 7.5-10.5 ml (Fig. 6A inset and data not shown). Quantification of TGF-β1 by ELISA indicated that prior to gel filtration rFBN1-N contained 1.41±0.17 ng/ml of active and 3.31±0.16 ng/ml of total TGF-β1, whereas TGF-β1 levels measured in eluted fractions containing rFBN1-N were below the detection limit of the ELISA (Fig. 6A). Based on standard globular proteins, we expect TGF-β1 to elute between 13-15 ml; however, TGF-β1 was not detected in the eluted fractions by ELISA. Therefore, to corroborate the ELISA readout, a cell signaling assay was conducted with rFBN1-N prior to and after gel filtration (Fig. 6B). Addition of rFBN1-N to fibroblasts triggered Smad2 phosphorylation, and activation of LAP-TGF-β1 by acidification stimulated stronger signaling. In contrast, gel-filtrated rFBN1-N did not trigger Smad2 phosphorylation even after activation by acidification.

To confirm that gel filtration can remove TGF-β1 from preparations of fibrillin-1 fragments irrespective of their size, rF23 was subjected to gel filtration chromatography (Fig. 6C, inset). Quantification of TGF-β1 by ELISA indicated that prior to gel filtration rF23 contained 1.9±0.2 ng/ml of total TGF-β whereas gel-filtrated fractions of rF23 (11–14 ml) contained TGF-β1 levels which were diluted below the detection limit of the ELISA (Fig. 6C). To confirm the ELISA results, a cell signaling assay was conducted with rF23 prior to and after gel filtration. Fractions eluting between 11-14 ml from gel filtration chromatography of rF23 were pooled, and exposed to fibroblasts (Fig. 6D). The cell signaling assay demonstrated that rF23 prior to gel filtration triggered Smad2 phosphorylation, and this activity was abolished in cell signaling assays conducted with gel-filtrated rF23.

The cell signaling assays conducted with gel-filtrated protein preparations of rF23 and rFBN1-N confirmed TGF-β1 quantification by ELISA, and demonstrated that non-specifically co-purified active and LAP-TGF-β1 can be removed by gel filtration chromatography under high salt conditions. However, the dissociated TGF-β1 dimer (25 kDa) and LAP-TGF-β1 dimer (100 kDa) were not detected in eluted fractions when analyzed by ELISA or AgNO_3_ staining (Fig. 6A, C, and data not shown). We speculate that TGF-β1 is not eluted because it interacts with the Superose 12 gel filtration resin. Our hypothesis is substantiated by Wakefield *et al.* who reported that active TGF-β1 is not readily eluted from the Superose 12 column [42]. Nevertheless, gel filtration chromatography is an efficient technique that can be used in conjunction with IMAC to further purify recombinant proteins.

### IMAC of Recombinant TGF-β1 and LAP

As TGF-β1 does not interact with the IMAC resin (Fig. 4), we hypothesized that TGF-β1 interacts with complexed nickel ions and thereby co-elutes with fractions containing protein contaminants and recombinant protein. Therefore, recombinant TGF-β1 and LAP (TGF-β1) were subjected to IMAC (Fig. 7). 1.5 µg of active TGF-β1 was loaded onto the IMAC column, and eluted fractions along with the flow-through were analyzed by ELISA (Fig. 7A). TGF-β1 was detected in fractions eluted with imidazole gradient between 9–16 ml (100–170 mM imidazole) with up to 2.28±0.04 ng of TGF-β1 detected in the peak fraction at 13.5 ml. TGF-β1 was not detected in the IMAC flow-through.

Similarly, 5 µg of LAP (TGF-β1) was loaded onto the IMAC column, followed by quantification of LAP present in the flow-through and eluted fractions by direct ELISA (Fig. 7B). LAP eluted under the imidazole gradient between 5–7 ml (40–65 mM imidazole), and was not detected in the IMAC flow-through.

Overall, although only 12% of active TGF-β1 and 9.8% LAP loaded onto the IMAC column were recovered, the data clearly demonstrate that TGF-β1 and LAP co-purifies with protein preparations by specifically interacting with nickel ions. Although it is not clear how exactly recombinant TGF-β1 and LAP interact with nickel, both proteins contain several clusters of histidine residues in exposed regions, which may mediate this interaction [43].

### Conclusions

Recombinant histidine-tagged extracellular proteins produced by HEK293 cells are routinely and conveniently purified by a one-step IMAC procedure. Quality controls of the purified proteins typically include SDS-PAGE, Western blotting and mass spectrometry. Here, we demonstrate using recombinant histidine-tagged fibrillin-1 fragments, that active and latent TGF-β1 co-purifies with these fragments by IMAC. This co-purification results from the interaction of active and latent TGF-β1 with the metal-loaded IMAC resin and not from direct interactions with the various fibrillin-1 fragments. Importantly, the amounts of TGF-β1 present in the protein preparations consistently triggered the canonical TGF-β1 signaling pathway in fibroblasts through Smad2 phosphorylation, whereas conventional methods did not detect TGF-β1 in the protein preparations. TGF-β1 could be effectively eliminated from the purified proteins by gel filtration under high salt conditions.

These findings are of significant importance, and their impact goes far beyond the production and purification of fibrillins by IMAC as this purification scheme is extensively used to generate secreted recombinant proteins, which are then subsequently used to examine cellular mechanisms. Based on the presented data, it is possible and even likely that a number of published experiments in the literature may be mis- or over-interpreted due to the potential and uncontrolled presence of either active or latent TGF-β1 in various secreted recombinant proteins after a one-step IMAC purification procedure. The results demonstrate the necessity of a rigorous quality control for histidine-tagged proteins purified by IMAC beyond the usual biochemical methods, if the purified protein will be used in relevant cellular analyses. In this study, we have demonstrated the presence of TGF-β1 in such protein preparations and excluded the presence of BMP-2. However, further analyses are required to determine if other potent cytokines and/or growth factors are co-purified with this chromatographic scheme.

## Supporting Information

Figure S1 **RT-PCR of transfected HEK293 clones to analyze the mRNA expression levels of TGF-β1.** The mRNA expression level of TGF-β1 among HEK293 cell clones transfected with various Fibrillin-1 fragments was analyzed by RT-PCR (40 cycles, 94°C, 60°C and 72°C; each for 1 min). The primer pairs for TGF-β1 (NM_000660.4) and GAPDH (NM_002046.4) were as follows: TGF-β1 (sense: 5'-CCCACAACGAAATCTATGACAAG-3'; antisense: 5'-CGGTGACATCAAAAGATAACCAC-3') GAPDH (sense: 5'-CCGCATCTTCTTTTGCGTCGC-3'; antisense: GACGGTGCCATGGAATTTGCC-3'). The expected product sizes are 258 bp for TGF-β1, and 226 bp for GAPDH. Note the expression of TGF-β1 in the rFBN1-C transfected HEK293 cell clone is lower compared to other stably transfected cell clones.
(PDF)
