# Supplementary material for: Immobilized Metal Affinity Chromatography Co-Purifies TGF-β1 with Histidine-Tagged Recombinant Extracellular Proteins
Source: PLoS One. 2012 Oct 31;7(10):e48629. doi: 10.1371/journal.pone.0048629 (PMC3485342; doi:10.1371/journal.pone.0048629)
Supplement: Figure S1 — RT-PCR of transfected HEK293 clones to analyze the mRNA expression levels of TGF-β1. The mRNA expression level of TGF-β1 among HEK293 cell clones transfected with various Fibrillin-1 fragments was analyzed by RT-PCR (40 cycles, 94°C, 60°C and 72°C; each for 1 min). The primer pairs for TGF-β1 (NM_000660.4) and GAPDH (NM_002046.4) were as follows: TGF-β1 (sense: 5'-CCCACAACGAAATCTATGACAAG-3'; antisense: 5'-CGGTGACATCAAAAGATAACCAC-3') GAPDH (sense: 5'-CCGCATCTTCTTTTGCGTCGC-3'; antisense: GACGGTGCCATGGAATTTGCC-3'). The expected product sizes are 258 bp for TGF-β1, and 226 bp for GAPDH. Note the expression of TGF-β1 in the rFBN1-C transfected HEK293 cell clone is lower compared to other stably transfected cell clones. (PDF) [file pone.0048629.s001.pdf]

Supplemental Figure S1

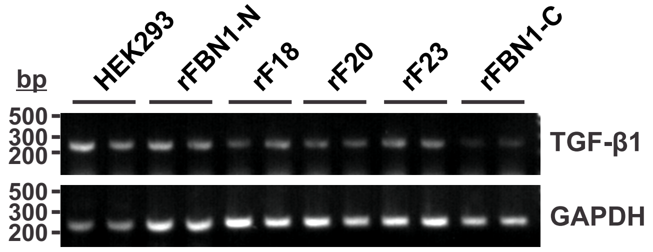

Supplemental Figure S1. RT-PCR of transfected HEK293 clones to analyze the mRNA expression levels of TGF-β1.

The mRNA expression level of TGF-β1 among HEK293 cell clones transfected with various fibrillin-1 fragments was analyzed by RT-PCR (40 cycles, 94°C, 60°C and 72°C; each for 1min). The primer pairs for TGF-β1 (NM_000660.4) and GAPDH (NM_002046.4) were as follows: TGF-β1 (sense: 5'-CCCACAACGAAATCTATGACAAG-3'; antisense: 5'-CGGTGACATCAAAAGATAACCAC-3') GAPDH (sense: 5'-CCGCATCTTCTTTTGCGTCGC-3'; antisense: GACGGTGCC ATGGAATTTGCC-3'). The expected product sizes are 258 bp for TGF-β1, and 226 bp for GAPDH. Note the expression of TGF-β1 in the rFBN1-C transfected HEK293 cell clone is lower compared to other stably transfected cell clones.
